# Nursing students’ experiences of developing spiritual care competencies through simulation: a qualitative exploratory descriptive study

**DOI:** 10.1186/s12912-026-04778-7

**Published:** 2026-05-21

**Authors:** Jordina Domènech-Sorolla, María Dolores Fernández-Pascual, Sara Pedregosa-Fauste, Daniel Medel, Fernando García-Díaz, Laura Martínez-Rodríguez

**Affiliations:** 1 Department of Nursing and Physiotherapy, University of Lleida, Carrer Montserrat Roig, 2, Lleida, 25198 Spain; 2 Grupo de Innovación Docente D-CIDES-Fundación Índex, Barcelona, Spain; 3 Department of Health Psychology, Faculty of Health Sciences, University of Alicante, Alicante, Spain; 4 Instituto de Investigación Sanitaria y Biomédica de Alicante (ISABIAL), Alicante, Spain; 5 Faculty of Nursing, University of Barcelona, Barcelona, Spain; 6 Grupo de Innovación Docente INTERMASTER, Universitat de Barcelona, Barcelona, Spain; 7 Grupo de Innovación Docente IDhEA-Fundación Index, Barcelona, Spain; 8 Departamento de Filosofía. IES La Madraza, Granada, Spain

**Keywords:** Spiritual care, Simulation, Nursing students, Experiential learning, Qualitative research, Holism

## Abstract

**Background:**

Spiritual care is a core component of holistic nursing; however, nursing students frequently report limited preparation and confidence in addressing patients’ spiritual needs. Although simulation-based education has shown promise for developing complex professional competencies, little is known about how nursing students experience the process of learning spiritual care through high-fidelity simulation. Therefore, this study aimed to explore nursing students’ perceptions and experiences regarding the development of spiritual care competencies through high-fidelity clinical simulation.

**Methods:**

The design of the study was qualitative phenomenological study. First-year undergraduate nursing students (*n* = 55) from a public Spanish university participated in a structured high-fidelity simulation focused on spiritual care. Data were collected through reflective narratives written after the simulation, following Lederman’s debriefing model. Data were analyzed using reflexive thematic analysis based on Braun and Clarke’s approach, combining inductive and deductive coding strategies and supported by ATLAS.ti software.

**Results:**

Four themes were identified: (1) emotional positioning in relation to spirituality, reflecting initial emotional responses to a novel learning experience; (2) negotiating complexity in spiritual care practice, comprising two subthemes: (2.1) relational uncertainty and ethical tension in spiritual encounters, and (2.2) relational enablers and communicative attunement in spiritual care; (3) meaning-making and reflective transformation, representing the development of reflective capacity and a deeper understanding of spiritual care as part of holistic nursing; and (4) integration of spiritual care into professional identity, highlighting perceived transferability to future clinical practice. Participants described simulation as a psychologically safe environment that promoted emotional engagement, ethical awareness, and reflective learning.

**Conclusion:**

High-fidelity simulation shows potential as an educational strategy for developing spiritual care competencies in nursing education. Introducing spiritual care training early in nursing curricula may enhance students’ ability to provide holistic, person-centered care that addresses patients’ spiritual needs.

**Clinical trial number:**

Not applicable.

**Supplementary Information:**

The online version contains supplementary material available at 10.1186/s12912-026-04778-7.

## Background

Nurses have a professional responsibility to provide spiritual care as a fundamental component of holistic care. Holistic care involves recognizing and responding to individuals’ biological, psychological, social, and spiritual needs in an integrated manner [1, 2]. Despite increasing recognition of spirituality as a core dimension of nursing practice, spiritual care remains inconsistently integrated into everyday healthcare practice, and substantial gaps persist in nurses’ knowledge, confidence, and practical skills for identifying and addressing patients’ spiritual needs. Recent literature indicates that nurses frequently feel unprepared and undertrained to provide spiritual care, even as increasingly diverse patient populations present complex spiritual concerns into clinical encounters, highlighting the need for structured education and competency frameworks to address these gaps. Educational frameworks such as the EPICC Spiritual Care Education Standard have been developed precisely to guide the integration of spiritual care competencies in nursing curricula, as nursing professionals worldwide continue to report uncertainty and variability in spiritual care provision in practice [3, 4].

To address this challenge, it is essential to educate nurses in spiritual care competencies that enable them to recognize spiritual distress, establish meaningful therapeutic relationships, communicate sensitively about spiritual concerns, and provide person-centered responses aligned with patients’ values and beliefs [3, 5]. The development of spiritual care competencies is closely linked to nurses’ intrapersonal awareness and reflection on their own spirituality, which has been shown to influence their ability to engage authentically and respectfully with patients’ spiritual needs [6, 7].

Education in spiritual care competencies is particularly important during undergraduate nursing training, as it lays the foundation for future professional practice. Strengthening these competencies has been associated with improved quality of care, enhanced patient well-being, and better physical, mental, and psychosocial health outcomes [8, 9]. Furthermore, integrating spiritual care competencies supports the development of humanized, person-centered nursing practice characterized by empathy, presence, effective communication, and respect for the whole person [10, 11].

Previous literature highlights the need for nursing students to strengthen their spiritual care competencies and for practicing nurses to increase awareness and actively integrate spiritual care into everyday clinical practice [7]. Consequently, nursing education programs are encouraged to adopt teaching strategies that explicitly promote the development of spiritual competencies at undergraduate level and within continuing professional education [10, 12]. Among available educational strategies, high-fidelity clinical simulation has emerged as a particularly promising methodology due to its experiential and reflective nature [11]. Simulation provides opportunities to practice complex competencies in a safe and controlled learning environment, facilitating reflection, clinical reasoning, and the integration of theory and practice [13, 14]. Several studies report that high-fidelity simulation enhances nursing students’ spiritual care competencies, self-confidence, and readiness for clinical practice, often exceeding the outcomes achieved through traditional teaching methods such as lectures or seminars [11, 14, 15]. Additionally, simulation-based education supports students in identifying and responding to patients’ spiritual needs within realistic clinical scenarios [16].

Although evidence supports the effectiveness of simulation-based learning for developing spiritual care competencies, existing literature predominantly emphasizes outcomes, perceived competence, or quantitative evaluations. Far less is known about how undergraduate nursing students experience the learning process itself when developing spiritual care competencies through high-fidelity simulation. In particular, there is limited understanding of how students interpret spiritual needs, navigate emotionally sensitive encounters, and construct meaning around spiritual care within simulated clinical contexts. This lack of qualitative evidence limits insight into the educational processes that underpin spiritual competency development.

To address this gap, this qualitative study aims to explore undergraduate nursing students’ perceptions, meanings, and lived learning experiences related to the development of spiritual care competencies through a high-fidelity clinical simulation intervention. This study forms part of a broader mixed-methods research project examining the effectiveness and educational impact of simulation-based learning on spiritual care competency acquisition.

## Methods

### Aim

To explore nursing students’ perceptions, meanings, and experiences of learning and developing spiritual care competencies through participation in high-fidelity clinical simulation.

### Study design

This study is framed within a qualitative phenomenological design aimed at exploring and describing individuals lived experiences of a particular phenomenon. Specifically, the phenomenon under study was the learning experience of undergraduate nursing students in developing spiritual care competencies through high-fidelity clinical simulation. This approach, based on the principles of naturalistic inquiry, allows for exploring how people perceive, interpret, and make sense of their experiences in their natural environment [17]. A phenomenological orientation was considered appropriate given the study’s focus on participants’ subjective meanings, interpretations, and sense-making processes related to spiritual care education. Its relevance lies in its ability to capture human experiences in depth, reveal essential meanings, and address complex phenomena from a holistic perspective.

### Sample

A purposive sampling strategy [18] was used, consistent with the phenomenological design of the study. A homogeneous sample of first-year undergraduate nursing students was selected, as all participants shared the common lived experience of participating in the same high-fidelity simulation activity. All reflective narratives submitted by eligible students were included in the analysis (*n* = 55). During data analysis, thematic saturation was observed after reviewing the majority of narratives, as no new codes or themes emerged in the final narratives analysed.

Participants were enrolled in the course *Evidence-Based Practice in Comprehensive Patient Care I* (150 h; 6 European Credit Transfer and Accumulation System [ECTS] credits) at a Spanish public university. Inclusion criteria were: (a) participation in the entire high-fidelity simulation activity, including submission of the reflective narrative; and (b) provision of written informed consent. No exclusion criteria were applied, as all students meeting the inclusion criteria were considered able to provide relevant experiential data.

### Development of high-fidelity simulation in spiritual care

Following the standards described by the International Nursing Association for Clinical Simulation and Learning (INACSL) [19], a high-fidelity simulation program was designed and implemented, following a structured and carefully planned chronology details the stages of developing the high-fidelity simulation designed for this study.

**Fig. 1:**
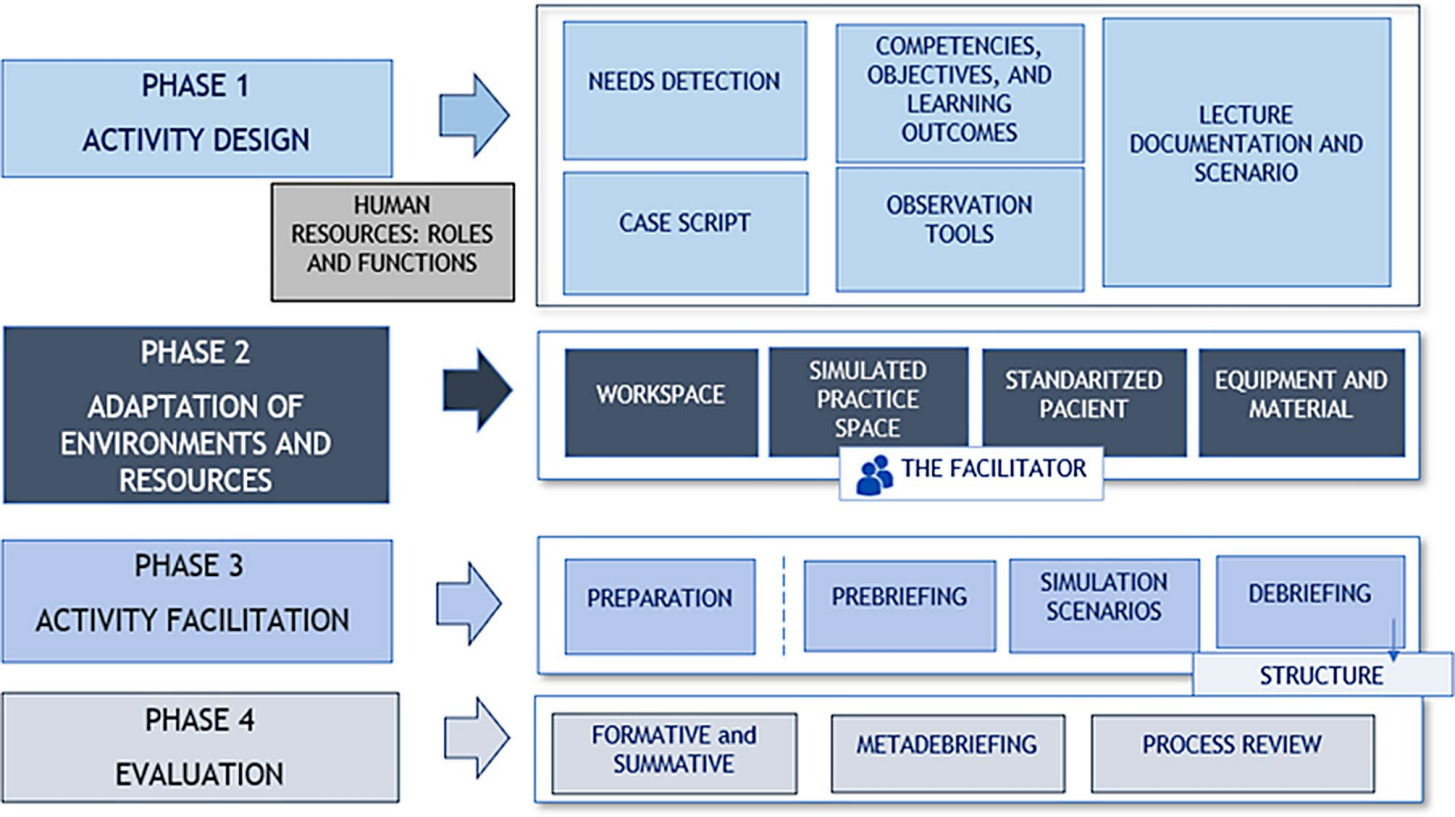
Stages of simulation program design according INACLS

In *Phase 1* of the activity design, the students’ learning needs in spiritual care were identified, and the desired learning objectives and outcomes were agreed upon collaboratively by the teaching team, including the content expert, simulation facilitators, and academic staff involved in the nursing program. The scenarios were created by a content expert in spiritual care. Spiritual care needs were incorporated into the design of each simulated scenario with the aim of achieving the established learning objectives according to INACSL standards [19]. The scenarios followed a chronological timeline focused on the same adult patient, admitted to a hospital ward and presenting with spiritual needs such as emotional distress, existential questioning, loss of meaning, and the need for compassionate presence and attentive listening, with spiritual needs that required nursing care.

In *Phase 2* of the activity design, the resources and environment were prepared, including the actor as a standardized patient, the setting, and the materials. To portray the patient protagonist of the case, a professional actor was used, considering that non-clinical skills are developed more effectively when interacting with a real actor rather than a mannequin [20]. This actor was an expert trained in clinical simulation cases, with extensive experience.

In *Phase 3*, the instructors specializing in spiritual care, together with the facilitator, carried out the different stages of the simulation: prebriefing, simulated scenarios, and debriefing. Each class group, consisting of (*n* = 40) students, was divided into medium-sized groups of (*n* = 10) students, and each subgroup of (*n* = 2–3) students carried out the simulated case according to each scenario. Each simulated scenario lasted 15 min, followed by a group debriefing with the medium-sized groups of (*n* = 10) students for 30 min, led by an advanced practice nurse specialized in spiritual care. Finally, in *Phase 4*, the activity evaluation process was carried out.

### Data collection

This qualitative study is part of a broader mixed-methods design corresponding to a doctoral thesis focused on evaluating a training intervention in spiritual competencies among nursing students. The quantitative phase was conducted using a pre-post design [11] employing the Spanish version of the Spiritual Care Competence Perception Scale (SSCRS-Sps) validated for nursing students [21]. For this qualitative study, individual reflective assignments completed by the participants after their participation in the spiritual care clinical simulation were collected. The reflective assignments were structured according to **Lederman’s debriefing model** [22], who considers guided reflection an essential component of experiential learning. This model outlines four fundamental phases: (1) objective description of the experience; (2) critical analysis; (3) generalization of learning; and (4) application to future situations. Its purpose is to promote deep reflection that allows transforming the experience into knowledge transferable to professional practice.

**Fig. 2:**
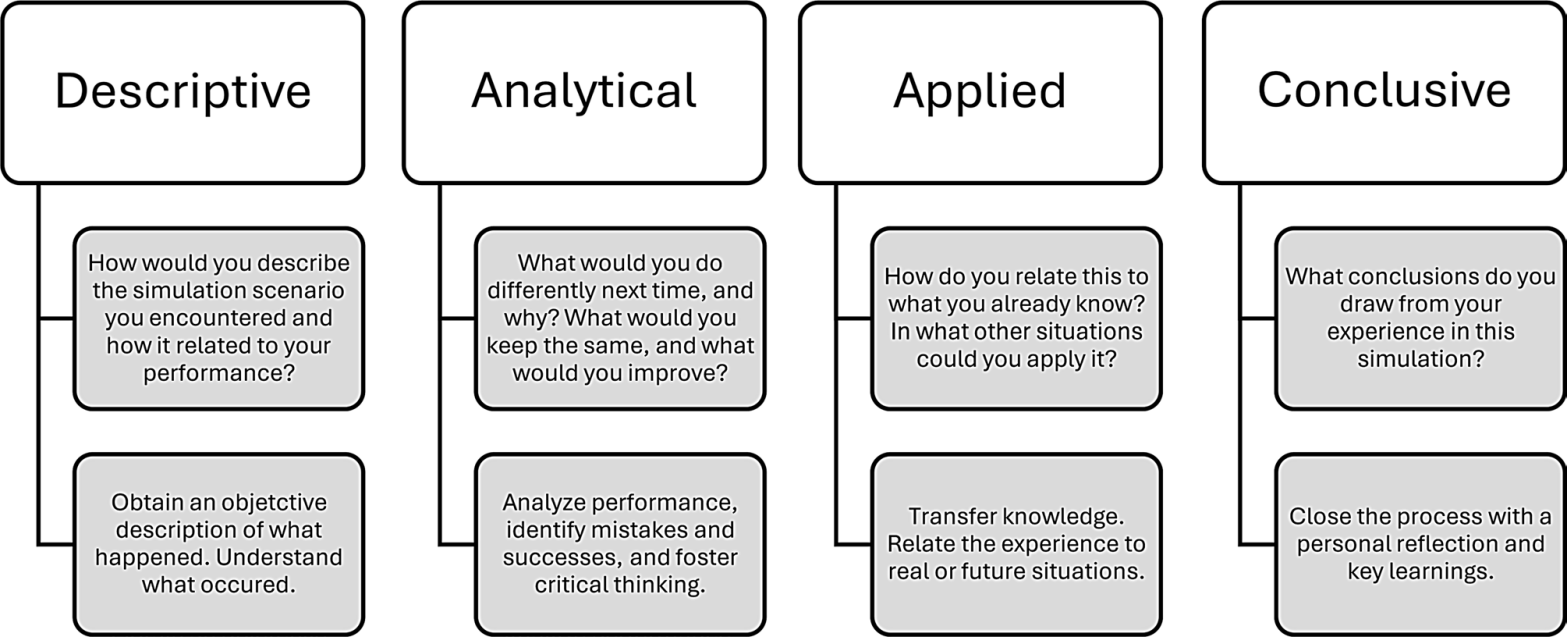
Structure of the reflective assignment. (Source: researchers’ own creation)

The reflective assignments completed by the participants were collected anonymously through a digital form made available on the university’s institutional OneDrive platform. There was no direct contact between the research team and the participants during data collection to ensure an ethical environment of trust and anonymity. This strategy aligns with methodological recommendations in qualitative research that advocate protecting participant autonomy and mitigating power imbalances in educational settings [23]. Data collection was conducted during May 2024. Fifty-five reflective journals were collected, each ranging from one to two pages per participant. The responses were organized in a spreadsheet and classified according to the structure of the assignment (description, analysis, application, and conclusion), which allowed for an initial systematization of the corpus.

### Data analysis

A thematic analysis was conducted following Braun and Clarke’s approach [24] to analyse the qualitative data. This technique made it possible to identify meaningful patterns and organize the reported experiences around recurring themes. To ensure the credibility and reliability of the analytical process, all phases of the analysis were carried out rigorously and sequentially: *(1) Familiarization with the data*: the transcripts of the reflective narratives were read in their entirety, recording initial impressions and key ideas; *(2) Initial coding*: the data were systematically coded using ATLAS.ti software, combining inductive coding (emerging from the data) with deductive coding aligned with the theoretical frameworks of the study;*(3) Searching for themes*: the most representative codes were grouped into initial themes in accordance with the process experienced by the participants during the simulation-based learning; *(4) Reviewing themes*: the internal coherence of each theme, its clear distinction from others, and its empirical support through quotations were examined; *(5) Defining and naming themes*: the final themes were defined and named, ensuring they captured the conceptual essence of the phenomenon analyzed; and *(6) Producing the report*: the results were presented using in vivo quotations to enhance the transferability of the findings [25]. The coding was reviewed by three members of the team, who independently analyzed the data and subsequently met to discuss interpretations and reach consensus.

### Rigor

This study was conducted following the guidelines established in the COREQ (Consolidated Criteria for Reporting Qualitative Research) checklist, ensuring methodological rigor and transparency throughout the process [26, 27] (See Supplementary material 1). The criteria of credibility, transferability, dependability, and confirmability were followed to ensure the quality of the study [28]. To ensure credibility, data triangulation was achieved through the involvement of three researchers, each contributing independent perspectives during the analysis process and participating in joint discussions to refine themes. Transferability was ensured through a thorough description of the context and participants’ characteristics. Dependability and confirmability were maintained through systematic data recording and collaborative review of analytic decisions by the research team throughout the analysis process.

### Ethical considerations

This project received approval from the Ethics Committee (CERT113) and the Data Protection Officer of the University of Lleida. Informed consent was obtained from all participants prior to the start of the study, and the confidentiality of the collected data was ensured [29]. Participation was voluntary, and the principal investigator securely stored the data. Students were informed that their participation or non-participation would have no impact on their course grades or their standing at the University.

## Results

The sample consisted of 55 students (*n* = 55), with a response rate of 100%. All participants were undergraduate nursing students, of whom were 90.9% female and 9.09% were male. The average age of the students was 19.2 years (See Table 1).

**Table 1:** Sample characteristics

Students’ characteristics	*n*	(%)
***Gender***		
Female	50	90.91
Male	5	9.09
***Age***		
17–18	27	40.09
19	9	16.36
20	9	16.35
> 20	10	18.18
x̄±SD 19.2 ± 2.04		
***Civil status***		
Single	48	87.27
Live with someone	7	12.73
***Number of children***		
0	55	100
***Level of studies completed***		
High school	40	72.73
CGS	14	25.45
Other degrees	0	0.00
Master	1	1.82

From the analysis of the reflective assignments, four main themes and two subthemes were identified. These themes represent an interpretive synthesis of participants’ experiences and learning processes, rather than a simple descriptive summary of their accounts. The themes aimed to explore, understand, and give meaning to participants’ experiences, perceptions, and learning in relation to the high-fidelity clinical simulation in spiritual care. The thematic structure reflects a chronological and experiential progression, aligned with the learning trajectory reported by participants during and after the simulation.

The first identified theme was (1) *Emotional positioning in the encounter with spirituality*, which captures participants’ immediate emotional responses when entering a scenario that was novel and emotionally demanding for many. The second theme, (2) *Negotiating complexity in spiritual care practice*, reflects how participants navigated obstacles and enabling factors while attempting to respond to patients’ spiritual needs. This theme illustrates the interplay between perceived challenges and the resources participants mobilized to manage them, highlighting how learning occurred through engagement with both difficulty and support. The third theme, (3) *Meaning-making and reflective transformation*, refers to the development of participants’ reflective capacity and their evolving understanding of spiritual care as a core dimension of holistic nursing. This theme reflects a shift from task-oriented action towards deeper meaning-making and self-awareness. The fourth theme, (4) *Embodied integration of spiritual care into professional identity*, represents participants perceived transferability of spiritual care competencies to future professional practice (see Table 2). A detailed description of the codes and illustrative quotations for each theme and subtheme is provided in Supplementary Material 2.

**Table 2:** Themes, subthemes, and definitions

Theme	Subthemes	Definitions
1: Emotional positioning in the encounter with spirituality		Captures the students’ initial perceptions upon entering the scenario and their immediate feelings.
2: Negotiating complexity in spiritual care practice	2.1 Relational uncertainty and ethical tension in spiritual encounters	This theme explores how students applied their knowledge, skills, and strategies to address a specific situation, analyzing how their responses were influenced by the challenges they faced and the factors that facilitated their learning.
	2.2 Relational enablers and communicative attunement in spiritual care	
3: Meaning-making and reflective transformation		This theme is based on a deepening understanding of the meaning of spiritual care as an essential nursing competency.
4: Embodied integration of spiritual care into professional identity		This theme explains how the knowledge and skills acquired through simulation are projected onto future professional contexts, demonstrating a high degree of transferability and meaningfulness of learning in the students’ training.

### Theme 1: Emotional positioning in the encounter with spirituality

Participants reported diverse emotional responses when engaging in the high-fidelity simulation in spiritual care. Some participants expressed feelings of insecurity, fear, discomfort, or a sense of “being lost” at the beginning of the simulation. These emotional reactions were interpreted as reflecting uncertainty related to addressing spirituality, a topic perceived as abstract, sensitive, and insufficiently explored in prior training. However, most participants reported feeling progressively more comfortable during the simulation, particularly due to the presence of a real actor and the realism of the scenario, which resembled situations encountered or anticipated in clinical placements.

Participants highlighted the importance of engaging in the simulation within a safe learning environment where mistakes were permitted and viewed as learning opportunities. This sense of psychological safety appeared to facilitate emotional engagement and openness to learning.

Participant quotations are identified using the code S (student) followed by a number (e.g., S6). As one participant stated: S6: *“Regarding the scenario*,* I would say that I felt comfortable in some aspects; it is true that knowing in advance what the situation was like and how the patient expressed himself made me feel more at ease.”* Other participants described initial anxiety and uncertainty when approaching the encounter. S8 stated: *“I was afraid I wouldn’t know what to say if the patient asked something I couldn’t answer.”* Likewise, S14 reported: *“I was really nervous at the beginning because I didn’t want to say the wrong thing or make the patient feel worse.”*

Despite these initial concerns, some students described personal growth and satisfaction after the experience. As S9 reflected: *“I didn’t think I could handle such an emotional situation*,* but in the end I felt proud of how I managed to stay present and supportive.”* These accounts suggest that emotional discomfort gradually evolved into confidence and engagement through participation in the simulation.

### Theme 2: Negotiating complexity in spiritual care practice

Participants reported that the simulation enabled them to identify both barriers and facilitating factors in addressing patients’ spiritual needs. This theme highlights the dynamic tension between difficulty and learning, illustrating how challenges acted as catalysts for reflection and skill development. Two subthemes were identified (2.1) relational uncertainty and ethical tension in spiritual encounters and (2.2) relational enablers and communicative attunement in spiritual care.

**Fig. 3:**
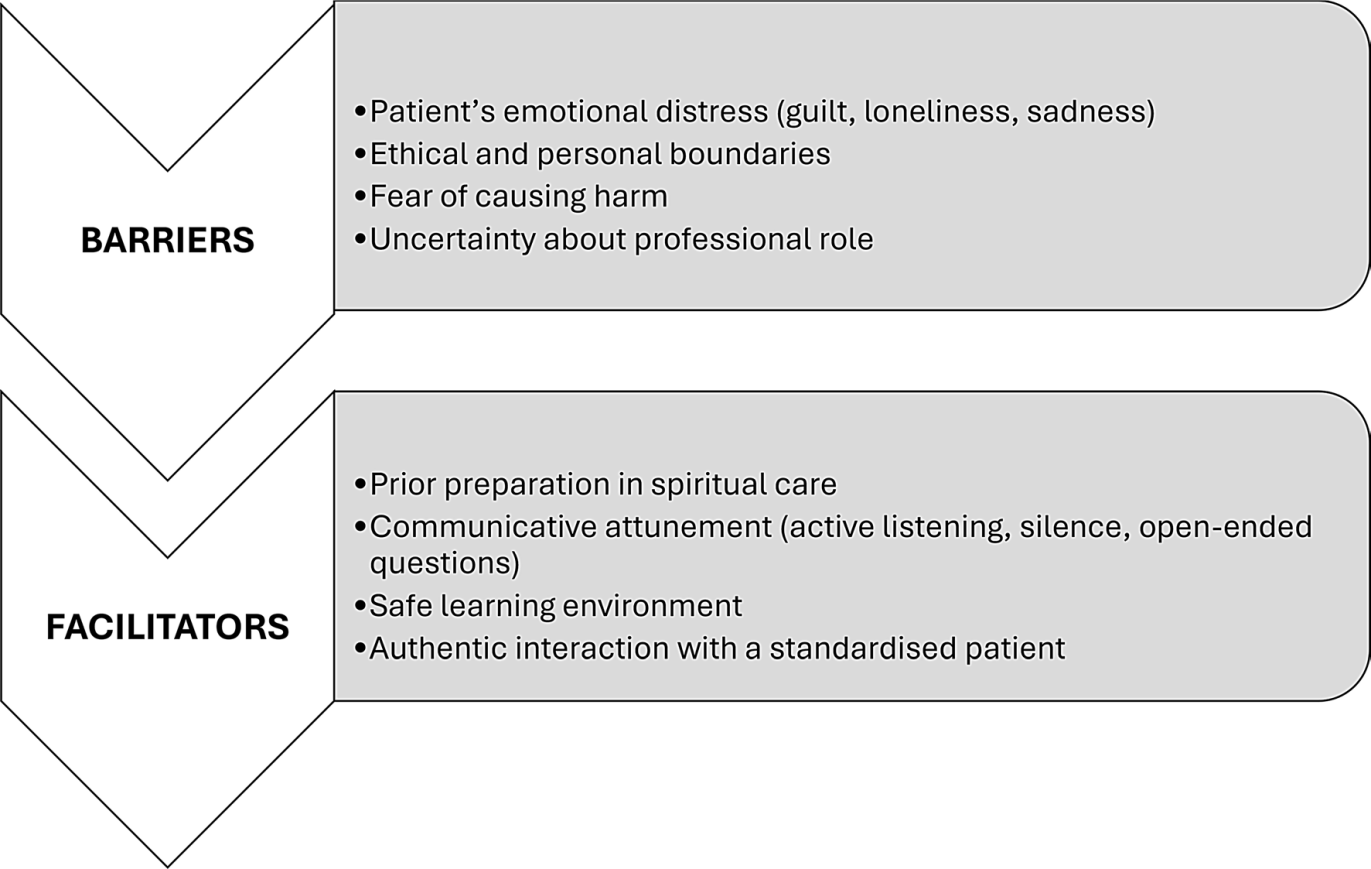
Representative barriers and facilitators of theme 2. (Source: researchers’ own creation)

#### Subtheme 2.1: Relational uncertainty and ethical tension in spiritual encounters

Participants acknowledged difficulties in establishing a trusting and empathetic relationship necessary to explore spiritual needs. These difficulties were interpreted as reflecting relational uncertainty when engaging with spirituality as an intimate and value-laden dimension of care. The barriers identified were primarily linked to discomfort with intimacy, fear of causing harm, and uncertainty about professional boundaries. Participants described challenges when patients expressed guilt, loneliness, or existential distress, highlighting a perceived lack of confidence in how to respond in ways that were both supportive and professionally appropriate.

This is illustrated in the following account: S3: *“The nurse must ensure trust and well-being through care*,* creating an atmosphere of affection and empathy where the patient feels safe and is able to express their spiritual needs; the nurse must be present at the right moment*,* listen*,* and accompany the patient*,* demonstrating concern and a genuine desire to help”.* S7 added a critical ethical dimension: *“We must not forget the ethical aspects of patients’ rights to hold their beliefs without having to disclose them.”* Other participants expressed uncertainty about how to respond without overstepping professional boundaries. S17 stated: *“I have to admit that it was very difficult for me to apply ethics properly in that moment. I didn’t know exactly how to approach the situation without imposing my views.”* Similarly, S5 described communicative resistance from the patient: *“The patient was very closed off and was not very participative as we asked questions. This created a tense and forced atmosphere during communication.”*

Taken together, these reflections suggest an emerging awareness of ethical sensitivity, relational responsibility, and the need to balance presence with respect for autonomy when addressing spirituality in nursing care.

#### Subtheme 2.2: Relational enablers and communicative attunement in spiritual care

Participants identified several factors that facilitated their engagement with spiritual care during the simulation. Notably, prior theoretical preparation and exposure to the concept of spirituality were described as key enablers. Participants perceived preparatory learning as providing a cognitive and emotional framework that reduced anxiety and increased confidence when initiating spiritually oriented conversations. S2 reported, *“Before starting to talk about how the simulation went*,* I want to emphasize that the two days prior were very important. I believe that reviewing the material covered in the theoretical class*,* along with an impromptu simulation*,* was very helpful in making me feel a bit more at ease on the day of the simulation.”* S21: *“This introduction process not only helped to establish a connection with the patient but also served as a tool to help me act more naturally and fully immerse myself in the scenario.”*

Participants also highlighted the importance of communication strategies, including the use of open-ended questions, respect for silence, avoidance of technical language, and active listening. These strategies were interpreted as forms of communicative attunement that enabled participants to recognize spiritual cues and respond in a person-centered manner. The realism of the scenario and interaction with the standardized patient further enhanced immersion and emotional engagement, reinforcing the perception of authenticity and learning relevance. In this regard, participants reflected on the importance of an active listening attitude, such as maintaining eye contact and engaging in the conversation on an equal level. Participants noted that this allowed them to assess the patient’s attitude and whether they were willing to engage in a conversation about spirituality. The students also reflected that they experienced the simulation as a real situation within a safe environment, as the realistic setting and the standardized patient helped them offer support and demonstrate emotional accompaniment.

Several quotations illustrated how communication and presence were perceived as therapeutic tools. S4 stated: *“As they spoke*,* I increasingly felt the patient’s need to be simply listened to and supported.”* Likewise, S11 reported: *“When the patient cried*,* I instinctively reached out and held their hand. It felt natural to offer comfort.”* In addition, S42 reflected that the realism of the scenario helped translate theory into practice: *“Being in that environment made the conversation feel genuine and helped me understand how spiritual care can be provided in real clinical settings.”*

### Theme 3: Meaning-making and reflective transformation

Participants reflected on spiritual care as an essential element of dignified, person-centered care. This theme represents a deepening of reflection, whereby participants moved beyond procedural understanding to a more value-based interpretation of spirituality. Spiritual care was described as supporting meaning-making, emotional well-being, and personal identity.

S21 stated, *“A spiritual need is defined as the need to maintain*,* strengthen*,* or recover one’s own beliefs.”* For this reason, they commented that: *“it is an important need*,* as it is what makes you a person and gives life meaning.”* Participants also recognized spirituality as an intimate and subjective dimension requiring respect and sensitivity. Reflection on boundaries and respect emerged as a significant learning outcome, particularly regarding avoiding intrusion or imposition. S8: *“Moreover*,* being there in the front row made me understand the meaning of spirituality*,* what the theory referred to*,* and how truly important it is. At times*,* I was left speechless listening to the patient and realizing the many spiritual needs they had.”* Participants also emphasized the importance of considering boundaries during the exploration of spiritual needs. Participants regarded spirituality as an intimate topic and, for some, even a taboo subject. Additionally, participants expressed that spirituality is subjective to each individual and must therefore be approached with great respect. S12: *“Spirituality requires respect*,* which the patient must be able to feel in order to deepen sufficiently and find solutions or purposes for improvement in the different aspects of the patient’s life.”* Participants also reflected on the emotional suffering underlying unmet spiritual needs. S7 stated: *“The patient expressed sadness while explaining that the only option they considered after learning about their amputation was to resort to euthanasia.”* Similarly, S11 reported: *“He told us he felt alone*,* and that he had even considered taking his life during his hospital stay.”* S4 added: *“He expressed regret about his past*,* saying his job had distanced him from his family and that he felt responsible.”*

Participants explicitly reported increased reflective capacity, noting greater awareness of silence, presence, empathy, and emotional attunement as core components of spiritual care. This suggests that simulation fostered reflective learning rather than solely technical skill acquisition.

### Theme 4: Embodied integration of spiritual care into professional identity

Participants analyzed how the simulation contributed to the development of spiritual care competencies applicable to future professional practice. They recognized spirituality as a legitimate patient need and valued learning strategies to address it within clinical contexts. Although the overall evaluation of the simulation was highly positive, some participants implicitly acknowledged initial discomfort and uncertainty, which gradually transformed into confidence through practice and reflection.

The simulation was perceived as a safe space for experiential learning, allowing participants to observe, practice, and learn from errors. This experiential integration reflects a perceived transfer of learning from the educational setting to anticipated professional roles, reinforcing holistic and person-centered approaches to care.

S4: *“As a future nurse*,* this simulation made me realize that I am in the right career and that it truly fulfills me. Health is the foundation of the human being*,* and nursing is capable of sustaining it.”*

Other participants described how the experience reshaped their understanding of nursing care. S33 stated: *“It made me see the importance of these moments and how they must not be missed in order to continue with standard medical intervention.”* Likewise, S52 reflected on future practice: *“In future clinical settings*,* I believe I’ll be more attentive to these silent needs that patients might not verbalize.”*

Some students also identified a curricular gap regarding spirituality in nursing education. S31 stated: *“No one had ever talked to us about how to detect or manage spiritual needs. This made me realize how neglected this dimension is in our education.”* These accounts suggest that the simulation not only strengthened professional identity but also increased awareness of the need for greater educational preparation in spiritual care.

## Discussion

The aim of this study was to explore the perceptions, meanings, and experiences of nursing students in developing spiritual competencies through clinical simulation. The findings provide in-depth insight into how high-fidelity simulation supports the emotional, relational, and reflective dimensions involved in learning spiritual care. The insights gained from this study highlight the importance of integrating spiritual competencies into nursing education [7]. The students who participated in the high-fidelity simulation in this study experienced an increased reflection on spirituality and acquired essential skills to identify and address patients’ spiritual needs. This process was characterized not only by skill acquisition but also by emotional positioning, ethical awareness, and reflective meaning-making, as reflected in the four themes identified. These results align with previous research suggesting that practical experience, both in simulated and real settings, enhances the ability for self-reflection and the integration of spirituality into healthcare [6, 14–16]. Spirituality has become established as an essential dimension of holistic care, especially in contexts of illness, suffering, or end-of-life care [30]. This dimension, which is deeply personal and existential, involves recognizing and addressing aspects related to life’s meaning, transcendence, and spiritual well-being. The results of this study illustrate how simulation-based learning enabled participants to engage simultaneously with emotional, psychological, relational, and spiritual aspects of care, thereby enacting a holistic approach consistent with core nursing principles. In this context, the training of healthcare professionals in spiritual competencies is essential to provide more humane, empathetic, and person-centered care. Recent studies have shown that incorporating spiritual scenarios in training environments, such as clinical simulation, facilitates the development of sensitive communication skills, fosters empathy, and promotes a deeper understanding of the patient’s spiritual suffering [13, 31, 32]. These educational experiences provide a safe space for reflection and learning, allowing students to explore their own stance on spirituality and consciously and respectfully adjust their approach [33].

A key aspect highlighted in the results of this study is the students’ perception of barriers and facilitators in spiritual care. Participants described relational uncertainty and ethical tension when engaging with patients’ spiritual needs, particularly in the presence of complex emotional states such as guilt, loneliness, and existential distress. The participants identified the importance of establishing a trusting and empathetic relationship with the patient, as well as employing communication strategies such as active listening, language adaptation, and managing silences. These findings underscore communicative attunement as a core spiritual care competency, and are consistent with previous studies that emphasize the relevance of effective communication in providing spiritual care [34]. However, the students also highlighted the difficulties associated with addressing complex emotional states such as guilt or loneliness, emphasizing the need for greater preparation to face these challenges [35]. This finding reinforces the importance of explicitly embedding emotional and ethical complexity within educational interventions aimed at developing spiritual care competencies.

The results also demonstrate that preparatory learning played a crucial role in facilitating students’ engagement with spiritual care during the simulation. Prior theoretical preparation, reflection on spirituality, and familiarization with communication strategies were perceived as reducing anxiety and increasing confidence when initiating spiritually oriented conversations. These findings respond directly to calls in the literature to better integrate preparatory activities with experiential learning in spiritual care education. Training in spiritual competencies therefore requires not only practical experiences but also adequate faculty preparation. The literature indicates that educators must be equipped to facilitate in-depth discussions on spirituality and guide students in integrating these competencies into their clinical practice [11, 36]. In this regard, curriculum design should incorporate specific programs that address spiritual care from a comprehensive approach, systematically combining theory and practice [6, 12].

Finally, the theme of embodied integration of spiritual care into professional identity highlights the perceived transferability of learning to future clinical practice. Participants described how experiential simulation supported the internalization of spiritual care as a legitimate and integral component of nursing practice, rather than an optional or abstract concept. There is a clear need to evaluate the long-term impact of spiritual care training on the clinical performance of future professionals. Recent studies have suggested that follow-up with students after training can provide valuable insights into the transfer of competencies to real-world practice [4, 37]. It is essential to compare different pedagogical approaches, such as clinical simulation versus other teaching methodologies, in order to identify the most effective strategies for acquiring competencies related to spiritual care. Similarly, it is necessary to explore how these competencies develop and are maintained over time, not only during academic training but also throughout professional practice, especially for nurses who face complex situations involving comprehensive patient care [6, 11].

## Limitations

This study presents some limitations that should be considered. First, it focused exclusively on the experiences of students from a single university, which may limit the transferability of the findings to other educational or cultural contexts. Second, the study was conducted at a single point in time, without subsequent follow-up, preventing observation of how spiritual competencies and the understanding of the phenomenon evolve over time. Future longitudinal studies would be valuable to examine the sustainability and transfer of spiritual care competencies into clinical practice. Third, the use of reflective written assignments as the sole qualitative data source may have influenced the depth and nature of participants’ accounts, as reflections are shaped by students’ writing abilities, self-awareness, and willingness to disclose personal experiences. Finally, although relevant elements regarding the experience during the simulation were identified, it remains necessary to further develop theoretical frameworks and tools that allow for a more systematic evaluation of the impact of these activities on learning spirituality in healthcare education, including dimensions such as knowledge patterns, competency progression, and curricular integration.

## Conclusions

This study is the first to provide an in-depth insight into the experiences and perceptions of nursing students regarding their formation in spiritual competencies through clinical simulation. The findings demonstrate that high-fidelity simulation supports not only technical and communicative learning but also emotional, relational, and reflective engagement with patients’ spiritual needs. Simulation emerges as a key methodology for developing spiritual competencies.

It is concluded that, from the first year of nursing education, it is possible to *“educate the gaze”* to promote a holistic approach to care that integrates biological, psychological, social, and spiritual dimensions. Through simulation, participants were able to practice addressing emotional and spiritual suffering alongside relational and ethical aspects of care, thereby enacting holistic nursing practice in a safe learning environment.

Notably, there is a need for greater emphasis on spiritual dimensions in nursing education and for providing guidelines to educators to strengthen training in this crucial aspect of holistic care. Embedding spiritual care within simulation-based learning can support the development of compassionate, person-centered nurses capable of responding to the full complexity of patients’ needs across clinical contexts.

## Electronic Supplementary Material

Below is the link to the electronic supplementary material.

Supplementary Material 1

Supplementary Material 2

## Data Availability

The datasets generated and/or analyzed during the current study are not publicly available as they form part of an ongoing doctoral thesis that has not yet been defended, but are available from the corresponding author on reasonable request. Supplementary files containing selected data are provided.

## Abbreviations

CERT: Comitè d’Etica Recerca i Transferència
COREQ: Consolidated Criteria for Reporting Qualitative Research
ECTS: European Credit Transfer and Accumulation System
EPICC: Spiritual Care Education Standard
INACSL: International Nursing Association for Clinical Simulation and Learning
SSCRS-Sps: Spiritual Care Competence Perception Scale
