# Supplementary material for: Nursing students’ experiences of developing spiritual care competencies through simulation: a qualitative exploratory descriptive study
Source: BMC Nurs. 2026 May 21;25:622. doi: 10.1186/s12912-026-04778-7 (PMC13366876; doi:10.1186/s12912-026-04778-7)
Supplement: Supplementary file 1 — Supplementary Material 1 [file 12912_2026_4778_MOESM1_ESM.docx]

| COREQ Checklist – Consolidated Criteria for Reporting Qualitative Research | Response |
| --- | --- |
| Domain 1: Research Team and Reflexivity |  |
| Personal Characteristics |  |
| 1. Interviewer/facilitator | Methods-Data collection |
| 2. Credentials | Title page |
| 3. Occupation | Title page |
| 4. Gender | Title page |
| 5. Experience and training | Title page |
| Relationship with Participants | Methods-Data collection |
| 6. Relationship established | Methods-Data collection |
| 7. Participant knowledge of the interviewer | Methods-Data collection |
| 8. Interviewer characteristics | Methods-Data collection |
| Domain 2: Study Design |  |
| Theoretical Framework |  |
| 9. Methodological orientation and theory | Methods-Study design |
| Participant Selection |  |
| 10. Sampling | Methods-Sample |
| 11. Method of approach | Methods-Data collection |
| 12. Sample size | Results – Sample characteristics |
| 13. Non-participation | Results – Sample characteristics |
| Setting |  |
| 14. Setting of data collection | Methods – Data collection |
| 15. Presence of non-participants | Methods – Data collection |
| 16. Description of sample | Results – Table 1 |
| Data Collection |  |
| 17. Interview guide | Methods – Data collection (Reflexive narratives & Lederman model) |
| 18. Repeat interviews | Not applicable |
| 19. Audio/visual recording | Not applicable |
| 20. Field notes | Not applicable |
| 21. Duration | Methods – Development of simulation |
| 22. Data saturation | Methods – Sample |
| 23. Transcripts returned | Not applicable |
| Domain 3: Analysis and Findings |  |
| Data Analysis |  |
| 24. Number of data coders | Methods – Data analysis |
| 25. Description of the coding tree | Methods – Data analysis & Supplementary material 2 |
| 26. Derivation of themes | Methods – Data analysis |
| 27. Software | Methods – Data analysis |
| 28. Participant checking | Not applicable |
| Reporting |  |
| 29. Quotations presented | Results & Supplementary material 2 |
| 30. Data and findings consistent | Results |
| 31. Clarity of major themes | Results |
| 32. Clarity of minor themes | Results |
