# Supplementary material for: Nursing students’ experiences of developing spiritual care competencies through simulation: a qualitative exploratory descriptive study
Source: BMC Nurs. 2026 May 21;25:622. doi: 10.1186/s12912-026-04778-7 (PMC13366876; doi:10.1186/s12912-026-04778-7)
Supplement: Supplementary file 2 — Supplementary Material 2 [file 12912_2026_4778_MOESM2_ESM.docx]

| Theme | Sub-theme | Code | Quotation |
| --- | --- | --- | --- |
| 1.Emotional positioning in the encounter with spirituality |  | Familiarity with the scenario increased emotional comfort | S6: *“Regarding the scenario, I would say that I felt comfortable in some aspects; it is true that knowing in advance what the situation was like and how the patient expressed himself made me feel more at ease.”*  *S20: “Although it was difficult, I felt good about being present in that room. It motivated me to want to learn more about the topic and improve my skills in spiritual support.* |
|  |  | Insecurity and nervousness before the experience | *S15: “Before starting to talk about how the simulation went, I want to emphasize that the two days prior were very important. I believe that reviewing the material covered in the theoretical class, along with an impromptu simulation, was very helpful in making me feel a bit more at ease on the day of the simulation.”*  *S8: “I was afraid I wouldn’t know what to say if the patient asked something I couldn’t answer.”*  *S14: “I was really nervous at the beginning because I didn’t want to say the wrong thing or make the patient feel worse.”*  *S9: “I didn’t think I could handle such an emotional situation, but in the end I felt proud of how I managed to stay present and supportive.”* |
| 2. Negotiating complexity in spiritual care practice | 2.1 Relational uncertainty and ethical tension in spiritual encounters | Awareness of the ideal relationship vs. difficulty achieving it in practice   \|  \| \| --- \|  \|  \| \| --- \| | S3: *“The nurse must ensure trust and well-being through care, creating an atmosphere of affection and empathy where the patient feels safe and is able to express their spiritual needs; the nurse must be present at the right moment, listen, and accompany the patient, demonstrating concern and a genuine desire to help.”*  S1: *"The spiritual dimension is a topic that, although it may not always seem so, is very taboo and intimate. That is why each person expresses themselves differently and talks about this subject within certain boundaries that must be respected. Our job as nurses is to listen and explore what possible solutions exist."* |
|  |  | Ethical tension in initiating spiritual dialogue | S7: *“We must not forget the ethical aspects of patients’ rights to hold their beliefs without having to disclose them.”*  *S17: “I have to admit that it was very difficult for me to apply ethics properly in that moment. I didn’t know exactly how to approach the situation without imposing my views.”* |
|  |  | Lack of training in spiritual care | S2: *“I did not have sufficient resources to properly address the situation and all I felt I could do was accompany the patient as best I could.”* |
|  |  | Patient’s resistance to openness | S5: “*The patient was very closed off and was not very participative as we asked questions. This created a tense and forced atmosphere during communication.”* |
|  | 2.2 Relational enablers and communicative attunement in spiritual care | Preparation prior to simulation enhances emotional readiness   \|  \| \| --- \|  \|  \| \| --- \| | *S15: “Before starting to talk about how the simulation went, I want to emphasize that the two days prior were very important. I believe that reviewing the material covered in the theoretical class, along with an impromptu simulation, was very helpful in making me feel a bit more at ease on the day of the simulation.”* |
|  |  | Introductory phase supports natural engagement and immersion | S21: *“This introduction process not only helped to establish a connection with the patient but also served as a tool to help me act more naturally and fully immerse myself in the scenario.”* |
|  |  | Creation of a safe environment | S12: *“The patient told us that we had helped them a lot, something inside was stirred to change their way of thinking and believe they could count on help.”*  S2: *“It made me see the importance of these moments and how they must not be missed in order to continue with standard medical intervention.”* |
|  |  | Active listening as a connection tool | S4: *“As they spoke, I increasingly felt the patient’s need to be simply listened to and supported.”*  *S11: “When the patient cried, I instinctively reached out and held their hand. It felt natural to offer comfort.”* |
|  |  | Empathy facilitating emotional release | S6: *“I told her that she was beautiful anyway, and that all I was doing was helping her so one day she could manage on her own. In the end, she hugged me and cried.”* |
| 3. Meaning-making and reflective transformation | – | Conceptual understanding of spiritual need | S21: *“A spiritual need is defined as the need to maintain, strengthen, or recover one’s own beliefs.”* |
|  |  | Emotional impact leading to deeper awareness of spirituality | S8: *“Moreover, being there in the front row made me understand the meaning of spirituality, what the theory referred to, and how truly important it is. At times, I was left speechless listening to the patient and realizing the many spiritual needs they had.”*  S7: *“He said working in the fields gave him a sense of peace and that it was what motivated him to keep going.”* |
|  |  | Respect as a foundation for meaningful spiritual care | S12: *“Spirituality requires respect, which the patient must be able to feel in order to deepen sufficiently and find solutions or purposes for improvement in the different aspects of the patient’s life.”* |
|  |  | Patient’s need to be heard | S7*: “The patient expressed sadness while explaining that the only option they considered after learning about their amputation was to resort to euthanasia.”*  S11: *“He told us he felt alone, and that he had even considered taking his life during his hospital stay.”*  S4: *“He expressed regret about his past, saying his job had distanced him from his family and that he felt responsible.”* |
| 4. Embodied integration of spiritual care into professional identity | – | Affirmation of vocational identity through experiential learning | S4: *“As a future nurse, this simulation made me realize that I am in the right career and that it truly fulfils me. Health is the foundation of the human being, and nursing is capable of sustaining it.”* |
|  |  | Shift in perspective about nursing role | S1: *“It made me see the importance of these moments and how they must not be missed in order to continue with standard medical intervention.”*  S4 *“As a future nurse, this simulation made me realize that I am in the right career and that it truly fulfils me. Health is the* *foundation of the human being, and nursing is capable of sustaining it.”* |
|  |  | Recognition of non-technical care as essential | S1: *“It made me see the importance of these moments and how they must not be missed in order to continue with standard medical intervention.”*  S6: *“In future clinical settings, I believe I’ll be more attentive to these silent needs that patients might not verbalize.”* |
|  |  | Human connection as a core of care | S20: *“Spiritual care is not about fixing things, but about being there. Sometimes, presence is the only intervention needed.”* |
|  |  | Realization of curricular gap | S9:*“No one had ever talked to us about how to detect or manage spiritual needs. This made me realize how neglected this dimension is in our education.”* |
